# AAPT Fibromyalgia Criteria and Concomitant Musculoskeletal Diagnoses in a Referral-Based Pain Clinic Cohort: A Retrospective Pilot Study

**DOI:** 10.3390/healthcare14203478

**Published:** 2026-10-16

**Authors:** Alessandro Casarella, Vincenzo Rania, Cristina Vocca, Gianmarco Marcianò, Riccardo Torta, Caterina Palleria, Valeria Antonazzo, Valentina Mastrangelo, Gian Pietro Emerenziani, Francesco Pio Oranges, Francesca Greco, Luca Gallelli

**Affiliations:** 1 Department of Health Sciences, Magna Graecia University of Catanzaro, 88100 Catanzaro, Italy; 2 Operative Unit of Clinical Pharmacology, Renato Dulbecco University Hospital, Viale Europa, 88100 Catanzaro, Italy; raniavincenzo1@gmail.com (V.R.); palleria@unicz.it (C.P.); 3 FAS@UMG Research Center, Department of Health Sciences, Magna Graecia University of Catanzaro, 88100 Catanzaro, Italy; 4 Department of Neurosciences ‘Rita Levi Montalcini’, University of Turin, Via Cherasco 15, 10126 Turin, Italy; riccardo.torta@unito.it; 5 Department of Primary Care, Azienda Sanitaria Provinciale, 88100 Catanzaro, Italy; antonazzo1982@gmail.com (V.A.); valentina.mastrangelo.vm@gmail.com (V.M.); 6 Department of Experimental and Clinical Medicine, University ‘Magna Græcia’ of Catanzaro, 88100 Catanzaro, Italy; emerenziani@unicz.it (G.P.E.); francescopio.oranges@studenti.unicz.it (F.P.O.); f.greco@unicz.it (F.G.)

**Keywords:** fibromyalgia, diagnostic criteria, AAPT criteria, diagnostic overlap, nociplastic pain, chronic widespread pain

## Abstract

Background/Objectives: Fibromyalgia (FM) is diagnosed using symptom-based criteria, and clinician-assigned labels may diverge from standardized classification frameworks. FM may also coexist with musculoskeletal and other pain-related conditions. We determined the proportion of patients in a combined referral-based cohort who fulfilled the 2019 ACTTION-American Pain Society Pain Taxonomy (AAPT) criteria after retrospective record-based adjudication and described documented concomitant musculoskeletal or pain-related diagnoses and symptom burden according to AAPT status. Methods: This retrospective, single-center cross-sectional pilot study included consecutive adult outpatients evaluated in a tertiary pain unit between January and April 2026 after referral for suspected FM or with a previous clinician-assigned FM diagnosis. AAPT classification was reconstructed from available longitudinal clinical documentation; the historical look-back varied by record availability and extended approximately 8–10 years in some patients. Results: Among 76 patients (96.1% female), 28 fulfilled the AAPT criteria (36.8%; 95% CI, 26.9–48.1). At least one documented concomitant musculoskeletal or pain-related diagnosis was recorded in 25/28 AAPT MET patients (89.3%; 95% CI, 72.8–96.3) and 26/48 AAPT NOT MET patients (54.2%), corresponding to a crude, unadjusted OR of 7.05 (95% CI, 1.87–26.54; *p* = 0.002). This association may partly reflect criterion-related overlap and differential diagnostic ascertainment. Exploratory analyses suggested greater symptom and affective burden in the AAPT MET group, whereas NRS pain intensity did not differ. Conclusions: Approximately one-third of this combined referral-based cohort fulfilled the AAPT criteria on retrospective adjudication. Documented concomitant musculoskeletal or pain-related diagnoses were common, but the observed association with AAPT status should not be interpreted as independent or causal. Standardized classification and multidimensional clinical pain phenotyping provide complementary information in chronic widespread pain.

## 1. Introduction

Fibromyalgia (FM) is a chronic pain disorder characterized by widespread musculoskeletal pain accompanied by fatigue, non-restorative sleep, cognitive dysfunction, and a broad spectrum of somatic and affective symptoms [1,2]. Affecting approximately 1–2% of the general population, it is among the most prevalent chronic pain conditions encountered in clinical practice [3,4]. Current evidence supports FM as the prototypical nociplastic pain condition, reflecting altered central pain processing in the absence of sufficient nociceptive or neuropathic mechanisms to fully explain symptom severity [1,2,5]. However, despite advances in the understanding of its pathophysiology, no validated laboratory, imaging, or molecular biomarkers are currently available. Consequently, the diagnosis of FM remains entirely clinical and depends on the application of standardized diagnostic criteria [1,5].

The diagnostic framework for FM has evolved considerably over the past three decades. The American College of Rheumatology (ACR) 1990 classification criteria [6] relied on widespread pain and tender-point examination, whereas subsequent revisions progressively shifted toward multidimensional symptom assessment through the Widespread Pain Index (WPI) and Symptom Severity Scale (SSS), culminating in the ACR 2010, 2011, and 2016 criteria [7,8,9]. More recently, the ACTTION-American Pain Society Pain Taxonomy (AAPT) [10] proposed simplified diagnostic criteria based on multisite pain together with clinically relevant fatigue or sleep disturbance. Although these criteria have improved the standardization of FM diagnosis for both research and clinical practice, their implementation remains heterogeneous. Consequently, discrepancies between clinician-assigned diagnoses and standardized diagnostic criteria have been consistently reported across primary-care and specialist settings [11,12,13,14,15], indicating that a previous diagnosis of FM does not necessarily correspond to fulfilment of contemporary diagnostic criteria. The diagnostic challenge is further complicated by the frequent coexistence of FM with other painful conditions. Nociplastic pain mechanisms may coexist with nociceptive pain arising from degenerative, inflammatory, or other musculoskeletal disorders, resulting in mixed pain phenotypes rather than mutually exclusive pain mechanisms [1,5]. Likewise, concomitant FM has been described across several rheumatic diseases, where it may amplify symptom burden, influence patient-reported outcomes, and complicate the interpretation of disease activity measures [16]. Accordingly, fulfilment of standardized FM criteria should not preclude systematic assessment for documented concomitant musculoskeletal or other pain-related conditions that may contribute to the overall clinical presentation [11,12].

Although discrepancies between clinician-assigned diagnoses and standardized criteria have been documented in primary-care and rheumatology populations, comparable evidence from specialist pain services remains scarce. This represents an important knowledge gap because pain clinics routinely evaluate patients with complex, multifactorial, and overlapping pain syndromes, in whom classification of fibromyalgia and characterization of coexisting pain mechanisms may both influence clinical reasoning and management. In particular, the proportion of patients in a combined referral-based cohort who fulfil the AAPT 2019 criteria after specialist record-based assessment remains poorly defined, as does the frequency of documented concomitant musculoskeletal or pain-related diagnoses among those meeting these criteria.

We therefore conducted an exploratory, single-center retrospective cross-sectional study of consecutive adult outpatients referred to a specialist pain unit for suspected FM or carrying a previous clinician-assigned diagnosis of FM. The primary objective was to determine the proportion of this combined referral-based cohort fulfilling the AAPT 2019 criteria following retrospective record-based specialist adjudication. Secondary objectives were to evaluate the frequency of documented concomitant musculoskeletal or pain-related diagnoses according to AAPT status and to describe symptom burden, affective symptoms, and pain characteristics between patients fulfilling and not fulfilling the AAPT criteria. The 36.8% fulfilment estimate was not intended as a diagnostic confirmation rate among previously diagnosed patients. As a pilot study, the findings are intended to generate hypotheses and provide preliminary estimates to support future prospective multicenter investigations.

## 2. Materials & Methods

### 2.1. Study Design and Population

This was a single-center retrospective cross-sectional study based on routinely collected clinical records from consecutive adult outpatients evaluated at the Pain Medicine Unit, Clinical Pharmacology Operative Unit, “Renato Dulbecco” University Hospital (Catanzaro, Italy), between January and April 2026. This interval represents the index inclusion period. Because the study was retrospective, review of clinical information was not restricted to this four-month period: available longitudinal documentation, including previous clinical history, symptom descriptions, specialist assessments, diagnostic investigations, and prior diagnoses, was reviewed when available. The retrospective look-back therefore varied among patients and extended approximately 8–10 years in some cases.

Patients were eligible if they had been referred by a physician for specialist evaluation of suspected fibromyalgia (FM) or had a previous clinician-assigned diagnosis of FM at the time of assessment. Both pathways represented physician-initiated specialist evaluation and were analysed as a single combined referral-based cohort because the retrospective referral documentation did not allow reliable stratification of the two pathways for all patients. Accordingly, the study was not designed to estimate diagnostic concordance specifically among patients with a previous FM diagnosis. Self-diagnosed presentations were not included.

All consecutive adult outpatients meeting the eligibility criteria during the study period were screened. No a priori clinical exclusion criteria were applied other than availability of sufficient clinical information for reliable AAPT adjudication. Of 81 records screened, five were excluded because the available documentation was insufficient to reconstruct the minimum information required for reliable AAPT classification and extraction of the study dataset; these exclusions were unrelated to inter-rater disagreement. Seventy-six patients were therefore included in the final analysis. As an exploratory pilot study, no formal a priori sample-size calculation was performed; all analyzable consecutive patients were included to provide preliminary estimates for future prospective studies.

### 2.2. Retrospective AAPT Adjudication

Because routine clinical records had not been specifically designed for standardized AAPT classification, AAPT status was reconstructed retrospectively from the available clinical documentation. Relevant information was derived from narrative medical history, descriptions of pain distribution, symptom documentation, clinical examination, and longitudinal records when available. The purpose of this process was to determine whether the available documentation supported the published 2019 AAPT criteria; it was not intended to recreate a prospective standardized examination.

For each patient, both assessors evaluated whether the available AAPT-relevant clinical information supported: (i) multisite pain involving at least six of the nine AAPT body regions; (ii) fatigue or sleep disturbance of at least moderate clinical relevance; and (iii) symptom persistence for at least three months. Pain-region involvement was determined from explicit anatomical descriptions in the clinical history and examination rather than from imaging abnormalities alone. Because routine records did not contain a standardized retrospective severity score specifically designed for AAPT adjudication, “moderate” fatigue or sleep disturbance was not reconstructed numerically; assessors relied on the clinician-documented description of symptom severity and clinical relevance. When documentation was too limited or ambiguous to support a reliable determination of the required components, the record was considered insufficient for AAPT classification.

AAPT adjudication was performed independently by two clinical investigators. No separate formal calibration exercise was performed; both assessors applied the same published AAPT 2019 framework and study definitions to the available records. During patient-level adjudication, the assessors did not have access to each other’s classification and were blinded to the referral or previous fibromyalgia label, documented concomitant musculoskeletal or pain-related diagnoses, FIQR, Zung SAS/SDS and DN4 scores, pharmacological treatment, and predefined comorbidity variables. Complete agreement was observed for the 76 classifiable records (76/76; 100% agreement; Cohen’s κ = 1.00). This agreement reflects reproducibility of interpretation of the available documentation and does not establish validity, completeness, or prospective equivalence of the source clinical information.

FIQR fatigue and sleep items were not available to the assessors during AAPT adjudication and were examined only after final AAPT status had been assigned. Accordingly, FIQR data could not alter AAPT classification. Because fatigue and sleep disturbance contribute conceptually to both AAPT fulfilment and overall fibromyalgia symptom burden, subsequent FIQR comparisons were treated as exploratory and interpreted with appropriate caution.

Patients were classified as AAPT MET when the available clinical information supported the AAPT 2019 criteria and as AAPT NOT MET when the criteria were not fulfilled. Records excluded for insufficient documentation were removed before the analytical phase and before calculation of inter-rater agreement.

The referral diagnosis or suspicion of FM defined the sampling frame and was considered distinct from the AAPT-based classification assigned during the study. Because suspected and previously diagnosed cases could not be reliably separated retrospectively for all patients, the AAPT fulfilment proportion applies only to the combined referral-based cohort. The operational framework used for retrospective AAPT adjudication, including the handling of insufficient or ambiguous documentation, is summarized in Supplementary Table S2.

### 2.3. Clinical Variables

Fibromyalgia-associated comorbid conditions were evaluated as a predefined set of eight conditions selected *a priori* on the basis of their frequent clinical association with fibromyalgia reported in the literature [17]. These included anxiety/depression, irritable bowel syndrome or other functional gastrointestinal disorders (including functional dyspepsia), migraine or chronic headache, sleep disorders (insomnia or obstructive sleep apnoea), thyroid disease, Raynaud’s phenomenon, psoriatic arthritis, and other autoimmune diseases. Each condition was coded as present or absent. The total number of these conditions defined the comorbidity count, whereas fibromyalgia-associated multimorbidity was defined as the presence of at least three predefined comorbid conditions. The prevalence of individual conditions is reported in Supplementary Table S1.

A documented concomitant musculoskeletal or pain-related diagnosis was defined as a clinician-recorded condition potentially capable of contributing to regional or widespread pain. In this cohort, these diagnoses consisted predominantly of degenerative, inflammatory, and other musculoskeletal disorders, including spinal osteoarthritis (spondyloarthrosis), polyarticular osteoarthritis (polyarthrosis), hip or knee osteoarthritis (coxarthrosis or gonarthrosis), psoriatic or rheumatoid arthritis, undifferentiated connective tissue disease, enthesitis, diffuse idiopathic skeletal hyperostosis, degenerative disc disease or disc herniation, and tendinopathy. Because ascertainment was retrospective and based on routine clinical documentation, diagnostic confirmation was not prospectively standardized across conditions; the records did not consistently permit a uniform requirement for imaging confirmation, specialist adjudication, or predefined examination criteria. These variables should therefore be interpreted as documented coexisting diagnoses rather than as uniformly validated causes of pain. Patients were coded dichotomously according to the presence or absence of at least one such diagnosis. Their presence did not preclude AAPT classification and was not considered evidence that the documented condition represented the sole or predominant pain mechanism. Differential intensity of diagnostic investigation may have introduced detection bias.

Because psoriatic arthritis and other autoimmune diseases contributed to both the fibromyalgia-associated comorbidity variable and the pain-related diagnosis endpoint, a post hoc sensitivity analysis recalculated fibromyalgia-associated multimorbidity after excluding these two overlapping categories. This analysis was exploratory and was performed to assess the robustness of the findings rather than to provide confirmatory evidence.

### 2.4. Data Extraction

Clinical characteristics, questionnaire data, and pharmacological information were manually extracted from the medical records and entered into the study database. All questionnaire scores were rechecked to verify scoring accuracy and data entry. Clinical records were also re-reviewed as part of the internal quality-control process to assess consistency between questionnaire data and the corresponding clinical assessment and diagnostic information documented in the chart. Comorbidities and medication classes were coded from the available clinical documentation. Questionnaire scores for the Revised Fibromyalgia Impact Questionnaire (FIQR), Zung Self-Rating Anxiety Scale (SAS), Zung Self-Rating Depression Scale (SDS), and Douleur Neuropathique 4 (DN4) questionnaire were obtained from completed instruments available in the clinical records [18,19,20,21].

### 2.5. Ethics

The study was approved by the Calabria Regional Ethics Committee (approval no. 110/2022, April 21). All patients provided general consent for the research use of anonymised clinical data. The study was conducted in accordance with the Declaration of Helsinki and is reported in accordance with the STROBE Statement for cross-sectional studies.

### 2.6. Statistical Analysis

Continuous variables are summarized as median and interquartile range (IQR), whereas categorical variables are presented as counts and percentages. Analyses were based on available-case data; no missing values were imputed, and the denominator is reported whenever observations were unavailable.

The primary analysis estimated the proportion of patients fulfilling the AAPT 2019 criteria among the 76 analyzed patients in the combined referral-based cohort. The key secondary outcome was the proportion of AAPT MET patients with at least one documented concomitant musculoskeletal or pain-related diagnosis. Ninety-five percent confidence intervals (95% CIs) for both proportions were calculated using the Wilson score method.

The frequency of the documented concomitant musculoskeletal or pain-related diagnosis endpoint was compared between AAPT MET and AAPT NOT MET patients using Fisher’s exact test, and the corresponding crude, unadjusted odds ratio (OR) with 95% CI was estimated. Because individual diagnoses were infrequent and not mutually exclusive, analyses were restricted to the predefined binary composite endpoint.

Fibromyalgia-associated multimorbidity, defined as the presence of at least three predefined fibromyalgia-associated comorbid conditions, was examined as a secondary exploratory outcome using Fisher’s exact test with estimation of the OR and 95% CI. Because psoriatic arthritis and other autoimmune diseases also contributed to the pain-related diagnosis endpoint, a post hoc sensitivity analysis repeated this comparison after excluding these two overlapping categories from the comorbidity count.

Variables reported in Tables 1, 3 and 5 are summarized descriptively by AAPT group, because the variables in these tables are either descriptive characteristics or categorized versions of continuous variables already compared on their original scale in Table 4. FIQR impact was categorized according to the predefined thresholds of mild (<39), moderate (39–58), and severe (≥59). Anxiety and depression severity were categorized using the established cut-offs for the Zung Self-Rating Anxiety Scale (SAS) and Self-Rating Depression Scale (SDS) based on raw summed scores.

In separate post hoc exploratory analyses, FIQR total score, Zung SAS and SDS raw scores, DN4 score, and pain Numerical Rating Scale (NRS) score were compared between AAPT MET and AAPT NOT MET patients using the Mann–Whitney U test. Variables reported in Supplementary Table S1 were compared using the Mann–Whitney U test for continuous variables and Fisher’s exact test for categorical variables. Across Table 4 and Supplementary Table S1, these analyses comprised 33 exploratory hypothesis tests in total. No multiplicity adjustment was applied. These analyses were conducted to characterize clinical patterns and generate hypotheses; individual *p* values should therefore not be interpreted as confirmatory evidence. For DN4 positivity, a post hoc missing-data sensitivity analysis examined the effect of possible allocations of the nine missing observations on the 2 × 2 comparison between AAPT groups; detailed allocations are reported in the Supplementary Material.

Because of the exploratory pilot design, modest sample size, and the number and heterogeneity of clinically plausible and partly overlapping covariates, no multivariable adjustment was performed. A post hoc multivariable model would have required data-driven covariate selection and could have produced unstable or overfitted estimates. Accordingly, all between-group effect estimates are crude and unadjusted and should not be interpreted as independent or causal associations.

All statistical analyses were performed using jamovi (version 2.7) with R (version 4.5).

### 2.7. Declaration of Generative AI Use

AI-based language tools (Claude 5, and ChatGPT-5, OpenAI) were used during manuscript preparation for English-language editing and internal consistency checks, including cross-checking numerical and statistical reporting.

The AI tools were not used for clinical adjudication, did not have access to directly identifiable patient information, and did not make autonomous scientific or clinical decisions. All analyses, interpretations, references, and final manuscript content were reviewed and approved by the authors, who take full responsibility for the work.

## 3. Results

### 3.1. Cohort and Primary Outcome

**Figure 1:**
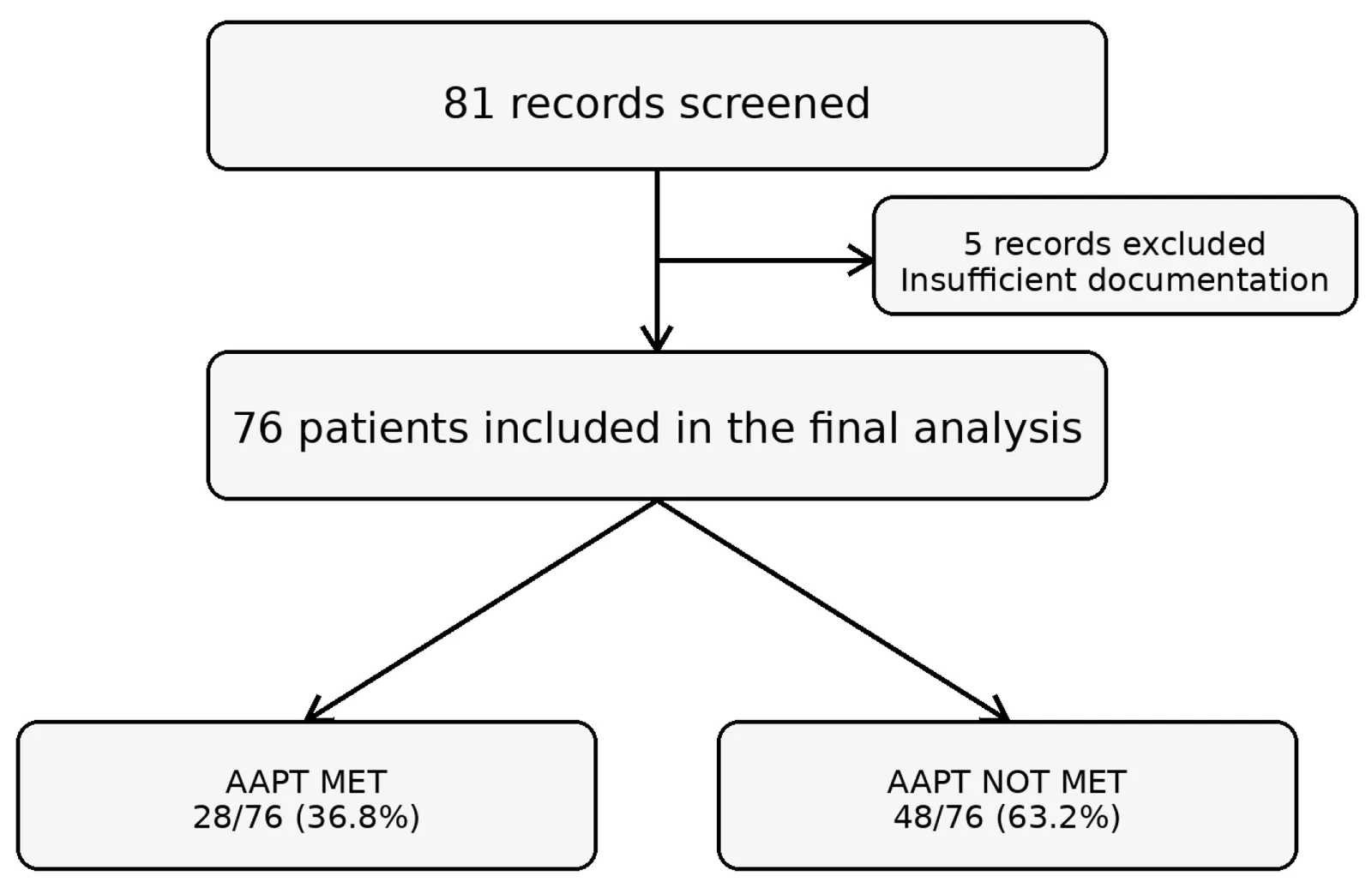
Study flow diagram. AAPT, ACTTION–American Pain Society Pain Taxonomy.

A total of 76 consecutive outpatients in the combined clinician-referred fibromyalgia pathway were included (median age 54 years [IQR 48.0–59.3]; 73/76, 96.1% female) (Table 1; Figure 1). Following retrospective record-based adjudication according to the AAPT 2019 framework, 28 of 76 patients were classified as AAPT MET (36.8%; 95% CI, 26.9–48.1) and 48 of 76 as AAPT NOT MET (63.2%) (Table 2). This proportion applies to the combined referral-based cohort and should not be interpreted as a diagnostic confirmation rate among previously diagnosed patients.

**Table 1:** Baseline demographic and clinical characteristics of the study cohort according to AAPT 2019 classification status.

Variable	Overall (N = 76)	AAPT MET (*n* = 28)	AAPT NOT MET (*n* = 48)
Female sex, *n* (%)	73 (96.1)	27 (96.4)	46 (95.8)
Age, years	54.0 [48.0–59.3]	52.0 [50.0–60.0]	54.5 [47.8–59.0]
BMI, kg/m^2^	25.6 [23.5–30.2]	26.4 [23.8–30.7]	24.7 [22.6–30.1]
Pain intensity (NRS, 0–10)	8.0 [6.0–9.0]	8.0 [7.0–9.0]	7.5 [6.0–9.0]
FIQR score (0–100)	51.4 [39.9–63.7]	58.7 [49.0–65.9]	45.3 [35.0–61.8]
Zung Anxiety Scale (SAS)	44.0 [36.8–52.3]	48.0 [41.0–54.8]	43.0 [33.5–48.3]
Zung Depression Scale (SDS)	43.0 [38.0–50.0]	46.5 [40.5–53.3]	41.0 [37.0–46.0]
Number of fibromyalgia-associated comorbidities	1.0 [0.0–2.0]	1.0 [1.0–3.0]	1.0 [0.0–1.3]
Positive DN4 screening (≥4), *n*/N (%)	48/67 (71.6)	22/24 (91.7)	26/43 (60.5)

Data are presented as median [interquartile range (IQR)] unless otherwise indicated. Categorical variables are expressed as *n* (%). This table is descriptive only; inferential comparisons are presented separately in Section 3. BMI values were available for 74 patients (26/28 patients fulfilling AAPT criteria and 48/48 patients not fulfilling AAPT criteria). DN4 positivity was calculated among patients with an available DN4 score (*n* = 67; 24 patients fulfilling AAPT criteria and 43 patients not fulfilling AAPT criteria). The number of fibromyalgia-associated comorbidities represents the sum (range, 0–8) of eight predefined comorbid conditions associated with fibromyalgia, as described in the Methods and reported individually in Supplementary Table S1. Abbreviations: AAPT, ACTTION–American Pain Society Pain Taxonomy; BMI, body mass index; DN4, Douleur Neuropathique 4 questionnaire; FIQR, Revised Fibromyalgia Impact Questionnaire; IQR, interquartile range; NRS, Numeric Rating Scale; SAS, Zung Self-Rating Anxiety Scale; SDS, Zung Self-Rating Depression Scale.

**Table 2:** Primary outcome and principal between-group comparisons.

Outcome	Estimate	Statistical Estimate
Patients fulfilling AAPT 2019 criteria (primary outcome)	28/76 (36.8%)	Wilson 95% CI: 26.9–48.1
Patients with a documented concomitant musculoskeletal or pain-related diagnosis among those fulfilling AAPT criteria (key secondary outcome)	25/28 (89.3%)	Wilson 95% CI: 72.8–96.3
Documented concomitant musculoskeletal or pain-related diagnosis: AAPT MET vs. AAPT NOT MET	25/28 (89.3%) vs. 26/48 (54.2%)	Crude, unadjusted OR 7.05 (95% CI: 1.87–26.54); Fisher’s exact test, *p* = 0.002

Data are presented as proportions unless otherwise indicated. Proportion estimates are accompanied by Wilson score 95% confidence intervals (CIs). Between-group comparisons were performed using Fisher’s exact test, and the effect size is expressed as a crude, unadjusted odds ratio (OR) with 95% CI. A documented concomitant musculoskeletal or pain-related diagnosis was defined as a clinician-recorded coexisting condition potentially capable of contributing to regional or widespread pain, as detailed in the Methods. The OR should not be interpreted as an independent or causal association. Abbreviations: AAPT, ACTTION–American Pain Society Pain Taxonomy; CI, confidence interval; OR, odds ratio.

### 3.2. Baseline Characteristics

In the overall cohort, median pain intensity on the Numeric Rating Scale (NRS) was 8/10 [IQR 6.0–9.0], and the median FIQR total score was 51.4 [IQR 39.9–63.7]. According to the predefined FIQR categories, 28/76 patients (36.8%) had severe disease impact, 30/76 (39.5%) moderate impact, and 18/76 (23.7%) mild impact; the distribution according to AAPT status is reported in Table 3.

**Table 3:** Distribution of Revised Fibromyalgia Impact Questionnaire (FIQR) severity categories according to AAPT 2019 classification status.

FIQR Severity Category	AAPT MET (*n* = 28)	AAPT NOT MET (*n* = 48)
Mild	1 (3.6%; 95% CI, 0.6–17.7)	17 (35.4%; 95% CI, 23.4–49.6)
Moderate	14 (50.0%; 95% CI, 32.6–67.4)	16 (33.3%; 95% CI, 21.7–47.5)
Severe	13 (46.4%; 95% CI, 29.5–64.2)	15 (31.3%; 95% CI, 19.9–45.3)

Data are presented as *n* (%) with Wilson score 95% confidence intervals (CIs). This table is descriptive only; no between-group hypothesis testing was performed. FIQR severity categories were defined using the prespecified thresholds: mild (<39), moderate (39–58), and severe (≥59). Abbreviations: AAPT, ACTTION–American Pain Society Pain Taxonomy; CI, confidence interval; FIQR, Revised Fibromyalgia Impact Questionnaire.

A positive DN4 neuropathic pain screening result (≥4) was present in 48/67 patients (71.6%) with an available score. FIQR total, Zung SAS, Zung SDS, and NRS were complete for all 76 analyzed patients, whereas DN4 was available for 67/76 (24/28 AAPT MET; 43/48 AAPT NOT MET) and BMI for 74/76 patients. Median raw Zung scores were 44 [IQR 36.8–52.3] for the Self-Rating Anxiety Scale (SAS) and 43 [IQR 38.0–50.0] for the Self-Rating Depression Scale (SDS). Patients had a median of 1 [IQR 0.0–2.0] fibromyalgia-associated comorbid condition. Complete descriptive characteristics for the overall cohort and by AAPT status are reported in Table 1 and Supplementary Table S1.

### 3.3. Documented Concomitant Musculoskeletal or Pain-Related Diagnoses

Among the 28 patients fulfilling the AAPT 2019 criteria, 25 (89.3%; 95% CI, 72.8–96.3) had at least one documented concomitant musculoskeletal or pain-related diagnosis, including spondyloarthrosis, polyarthrosis, psoriatic arthritis, undifferentiated connective tissue disease, or rheumatoid arthritis (Table 2). These diagnoses were chart-documented coexisting conditions and were not uniformly adjudicated as the principal cause of pain.

A documented concomitant musculoskeletal or pain-related diagnosis was recorded more frequently in AAPT MET than AAPT NOT MET patients (25/28, 89.3% vs. 26/48, 54.2%; Fisher’s exact *p* = 0.002; crude, unadjusted OR 7.05, 95% CI 1.87–26.54) (Table 2). Because this was an unadjusted retrospective comparison, the estimate should not be interpreted as an independent association and may partly reflect criterion-related overlap and differential diagnostic ascertainment.

### 3.4. Fibromyalgia-Associated Multimorbidity

Fibromyalgia-associated multimorbidity (≥3 predefined comorbid conditions) was present in 8/28 AAPT MET patients (28.6%) and 3/48 AAPT NOT MET patients (6.3%; Fisher’s exact *p* = 0.015; OR, 6.00; 95% CI, 1.44–25.01). However, after excluding psoriatic arthritis and other autoimmune diseases from the comorbidity count because these categories overlapped with the pain-related diagnosis endpoint, multimorbidity was present in 2/28 and 3/48 patients, respectively (OR, 1.15; 95% CI, 0.18–7.36; Fisher’s exact *p* > 0.999). Thus, the apparent multimorbidity association was not robust to removal of overlapping diagnostic categories and should be interpreted cautiously.

### 3.5. Symptom Severity According to AAPT Status

According to the predefined FIQR categories (mild < 39, moderate 39–58, severe ≥ 59), AAPT MET patients were classified as severe in 13/28 cases (46.4%; 95% CI 29.5–64.2), moderate in 14/28 (50.0%; 95% CI 32.6–67.4), and mild in 1/28 (3.6%; 95% CI 0.6–17.7). Among AAPT NOT MET patients, 15/48 (31.3%; 95% CI 19.9–45.3) were classified as severe, 16/48 (33.3%; 95% CI 21.7–47.5) as moderate, and 17/48 (35.4%; 95% CI 23.4–49.6) as mild (Table 3).

In post hoc exploratory analyses, AAPT MET patients showed higher FIQR total, Zung SAS, Zung SDS, and DN4 score distributions than AAPT NOT MET patients, whereas NRS pain intensity was similar between groups (Table 4). Because these comparisons were part of a broader set of 33 exploratory hypothesis tests without multiplicity adjustment, the corresponding *p* values are hypothesis-generating rather than confirmatory. DN4 scores were available in 67/76 patients (24/28 AAPT MET; 43/48 AAPT NOT MET); the median DN4 score was 5 in both groups, with different interquartile ranges. A separate post hoc sensitivity analysis of DN4 positivity (score ≥ 4) showed that the association between AAPT status and screening positivity was sensitive to the assumed allocation of the nine missing observations (Supplementary Analysis S1). DN4 is a screening instrument for neuropathic-like symptoms and signs and does not establish neuropathic pain or a lesion or disease of the somatosensory nervous system. Detailed missing-data sensitivity results are provided in the Supplementary Material.

**Table 4:** Symptom-severity scales by AAPT 2019 status.

Scale	AAPT MET (*n* = 28)	AAPT NOT MET (*n* = 48)	*p*
FIQR total (0–100)	58.7 [49.0–65.9]	45.3 [35.0–61.8]	0.013
Zung anxiety (SAS, 20–80)	48 [41.0–54.8]	43 [33.5–48.3]	0.004
Zung depression (SDS, 20–80)	46.5 [40.5–53.3]	41 [37.0–46.0]	0.017
DN4 (0–10) (*n* = 24/43)	5 [5.0–6.0]	5 [2.5–6.0]	0.033
Pain intensity, NRS (0–10)	8 [7.0–9.0]	7.5 [6.0–9.0]	0.116

Post hoc exploratory analysis. Data are presented as median [IQR] and were compared using the two-sided Mann–Whitney U test. These five tests were part of a broader set of 33 exploratory hypothesis tests across Table 4 and Supplementary Table S1; no multiplicity correction was applied. *p* values are presented for descriptive hypothesis generation and should not be interpreted as confirmatory evidence. DN4 was available in 67/76 patients (24 AAPT MET and 43 AAPT NOT MET) and is a screening instrument for neuropathic-like symptoms and signs rather than a diagnostic test for neuropathic pain. Abbreviations: AAPT, ACTTION–American Pain Society Pain Taxonomy; DN4, Douleur Neuropathique 4 questionnaire; FIQR, Revised Fibromyalgia Impact Questionnaire; IQR, interquartile range; NRS, Numeric Rating Scale; SAS, Zung Self-Rating Anxiety Scale; SDS, Zung Self-Rating Depression Scale.

According to the predefined Zung severity categories based on raw scores, fewer AAPT MET than AAPT NOT MET patients were classified within the normal range for anxiety (SAS: 2/28, 7.1% vs. 16/48, 33.3%) and depression (SDS: 7/28, 25.0% vs. 18/48, 37.5%). The complete distribution across severity categories is reported in Table 5.

**Table 5:** Anxiety and depression severity (Zung SAS/SDS) by AAPT 2019 status.

Severity Band	AAPT MET (*n* = 28)	AAPT NOT MET (*n* = 48)
Anxiety (Zung SAS)		
Normal	2/28 (7.1%) [2.0–22.6]	16/48 (33.3%) [21.7–47.5]
Mild–moderate	11/28 (39.3%) [23.6–57.6]	18/48 (37.5%) [25.2–51.6]
Marked–severe	12/28 (42.9%) [26.5–60.9]	13/48 (27.1%) [16.6–41.0]
Extreme	3/28 (10.7%) [3.7–27.2]	1/48 (2.1%) [0.4–10.9]
Depression (Zung SDS)		
Normal	7/28 (25.0%) [12.7–43.4]	18/48 (37.5%) [25.2–51.6]
Mild	8/28 (28.6%) [15.3–47.1]	19/48 (39.6%) [27.0–53.7]
Moderate	10/28 (35.7%) [20.7–54.2]	9/48 (18.8%) [10.2–31.9]
Severe	3/28 (10.7%) [3.7–27.2]	2/48 (4.2%) [1.2–14.0]

Descriptive analysis; no inferential testing. Data are presented as *n* (%) with Wilson 95% CIs. Severity was graded directly from raw summed scores using thresholds corresponding to conventional Zung index-score categories (index score = raw score × 1.25). SAS: <36 normal, 36–47 mild–moderate, 48–59 marked–severe, ≥60 extreme. SDS: <40 normal, 40–47 mild, 48–55 moderate, ≥56 severe. SAS, Zung Self-Rating Anxiety Scale; SDS, Zung Self-Rating Depression Scale.

### 3.6. Self-Reported Pharmacological Treatment

Self-reported pharmacological treatment patterns are presented descriptively in Supplementary Table S1. No clear between-group pattern emerged across individual medication classes. These data should be interpreted cautiously because reliable information on timing, dose, treatment duration, adherence, treatment indication, and distinction between current and previous use was not available from the retrospective records.

## 4. Discussion

In this retrospective real-world study of 76 consecutive outpatients in a combined referral-based fibromyalgia cohort, 28 patients (36.8%; 95% CI, 26.9–48.1) fulfilled the 2019 AAPT criteria after record-based specialist adjudication. This estimate applies to the combined population of patients referred for suspected FM or carrying a previous clinician-assigned FM diagnosis and should not be interpreted as a diagnostic confirmation rate among previously diagnosed patients. Referral or working diagnostic labels and AAPT classification therefore identified overlapping but non-identical clinical constructs in this setting.

The fulfilment rate observed in this cohort is numerically within the range reported in some previous studies examining agreement between clinician-assigned fibromyalgia labels and standardized criteria, but direct numerical comparisons are inappropriate because populations, referral pathways, settings, and diagnostic frameworks differ. Srinivasan et al. [13] evaluated the 2016 ACR criteria in primary care, whereas Cassisi and Sarzi-Puttini [11] evaluated a rheumatology referral population using the 2016 ACR framework. These studies are therefore most informative as evidence that clinician-assigned diagnoses and formal criteria do not identify identical populations, rather than as directly comparable fulfilment estimates.

Cassisi and Sarzi-Puttini [11] found that 41.2% of patients referred to a specialized rheumatology clinic with suspected or previously diagnosed fibromyalgia satisfied the 2016 ACR criteria. Their population included patients who personally suspected FM, while, in contrast, our cohort was restricted to physician-referred patients. Moreover, our cohort combined suspected and previously diagnosed FM cases and could not be reliably disaggregated into these referral pathways, which precludes interpretation of the 36.8% AAPT estimate as a measure of concordance among previously diagnosed patients. Differences in clinical setting and classification framework further limit direct cross-study comparison.

Population-based studies provide additional context but are even less directly comparable. Using data from the 2012 US National Health Interview Survey, Walitt et al. [14] estimated that nearly three-quarters of individuals reporting a clinician diagnosis of fibromyalgia did not fulfil surrogate criteria derived from the modified 2011 ACR framework. Agreement between clinician-assigned diagnosis and criteria-based classification has also been reported as only fair in a university rheumatology clinic (κ = 0.41) [15]. These studies support the broader concept that clinical labels and criteria-based classifications do not perfectly overlap, while differences in setting and case ascertainment preclude direct numerical equivalence with the present cohort.

Our findings add preliminary data from a European tertiary pain-unit population evaluated according to the 2019 AAPT framework and are consistent with the broader literature describing under-, over-, and misdiagnosis of fibromyalgia [12]. The very high proportion of women in the cohort (96.1%) should also be considered when interpreting external validity: it reflects the referral population studied but may limit generalizability of the 36.8% fulfilment estimate to more sex-balanced populations.

This distinction is clinically relevant because the AAPT and ACR frameworks use different case-definition structures. AAPT was designed as a concise clinically applicable framework centered on multisite pain together with clinically relevant fatigue or sleep disturbance, whereas the ACR framework incorporates a broader multidimensional assessment of pain distribution and symptom severity. Comparative studies therefore suggest that the operational simplicity of AAPT should not be equated with uniformly greater permissiveness. Differences in fulfilment rates across classification systems should be interpreted in light of their distinct structures rather than as evidence that one framework is intrinsically superior.

For example, Kang et al. [22] reported that 56.8% of patients with established fibromyalgia fulfilled the AAPT criteria compared with 94.7% using the 2016 ACR criteria, with lower sensitivity and high specificity for AAPT; Salaffi et al. [23] likewise demonstrated clinically relevant differences in case identification across AAPT and ACR frameworks. Therefore, failure to satisfy the AAPT framework should not be considered sufficient to exclude a clinical diagnosis of fibromyalgia in an individual patient, particularly when another validated framework may classify the same patient differently.

A second finding was the high prevalence of documented concomitant musculoskeletal or pain-related diagnoses among patients who fulfilled the AAPT criteria. Twenty-five of 28 AAPT MET patients had at least one such chart-documented condition, and the composite endpoint was more frequent in the AAPT MET group than in the AAPT NOT MET group (89.3% vs. 54.2%; crude, unadjusted OR 7.05; 95% CI, 1.87–26.54). This association should not be interpreted as evidence that musculoskeletal disorders independently predispose to AAPT-defined fibromyalgia. An important alternative explanation is structural overlap between the AAPT multisite-pain requirement and the pain distribution produced by polyarticular degenerative or inflammatory disease. Patients with greater symptom burden may also have undergone more extensive diagnostic evaluation, increasing the likelihood that coexisting structural abnormalities were documented. Referral bias, detection bias, and residual confounding therefore remain plausible explanations, and the cross-sectional design precludes causal interpretation.

The high prevalence of documented concomitant musculoskeletal or pain-related conditions is consistent with the recognized coexistence of fibromyalgia and chronic rheumatic disorders. Fibromyalgia may accompany inflammatory arthritis, osteoarthritis, and other chronic musculoskeletal diseases, in which nociplastic, nociceptive, and inflammatory mechanisms can coexist and contribute jointly to the pain experience [1,5,16]. The presence of a structural diagnosis therefore does not by itself establish the mechanism responsible for the patient’s overall pain burden.

Accordingly, the presence of a structural or inflammatory disorder should not be regarded as incompatible with fibromyalgia, just as fulfilment of fibromyalgia criteria should not preclude consideration of other clinically relevant pain mechanisms [10,24]. The multimorbidity sensitivity analysis further illustrates the importance of avoiding overinterpretation: the initial association disappeared after removal of overlapping diagnostic categories. Overall, these findings are best viewed as descriptive evidence of diagnostic and mechanistic complexity within this referral population rather than as evidence of independent etiological relationships.

The exploratory comparisons of symptom severity provided additional information regarding the clinical profile of patients fulfilling the AAPT criteria. Individuals in the AAPT MET group showed higher FIQR total scores together with higher Zung Anxiety Scale and Zung Depression Scale scores than patients who did not fulfil the criteria, whereas pain intensity measured by the Numerical Rating Scale did not differ significantly between groups (Table 4).

These symptom-severity comparisons were exploratory, post hoc, and unadjusted for multiplicity. Thirty-three exploratory hypothesis tests were performed across Table 4 and Supplementary Table S1, and isolated nominal *p* values may therefore represent chance findings. The results should be interpreted as patterns for hypothesis generation rather than as confirmatory evidence. FIQR data were not available during AAPT adjudication and could not influence classification; nevertheless, criterion-content overlap is expected because fatigue and sleep disturbance contribute both to AAPT fulfilment and to global fibromyalgia symptom burden [5,10,25].

The DN4 result warrants particular caution. Although the complete-case comparison of DN4 scores yielded a nominally low *p* value, the median score was identical in the two groups and nine observations were missing. A separate sensitivity analysis of DN4 positivity (score ≥ 4) showed that the association between AAPT status and screening positivity was sensitive to assumptions about the missing observations. Moreover, DN4 is a screening instrument for neuropathic-like symptoms and signs rather than a diagnostic test for neuropathic pain [26,27]. Accordingly, these data should not be interpreted as evidence that AAPT MET patients have a distinct neuropathic pain phenotype.

Taken together, these findings emphasize that fibromyalgia classification and pain-mechanism phenotyping are related but distinct clinical processes. Fulfilment of AAPT criteria identifies a symptom-defined syndrome but does not establish that all pain experienced by an individual patient is nociplastic. Nociplastic mechanisms may coexist with nociceptive pain related to degenerative musculoskeletal disease, inflammatory pain, and neuropathic-like symptoms. Recent work has likewise emphasized the clinical relevance of nociplastic symptom burden and the need to distinguish predominant nociplastic from mixed-pain phenotypes in fibromyalgia [28,29]. Standardized classification should therefore complement, rather than replace, comprehensive clinical assessment of individual pain mechanisms.

Pharmacological treatment was a secondary descriptive variable rather than a principal study objective. No clear between-group pattern was observed across medication classes, but interpretation is limited by the retrospective quality of medication histories and the absence of reliable information on timing, dose, duration, adherence, indication, and current versus previous use. These data are therefore retained primarily for descriptive transparency in Supplementary Table S1.

### Limitations

This study has several limitations that should be considered when interpreting the findings.

First, this was a single-center retrospective cross-sectional pilot study based on a relatively small tertiary pain-unit cohort. The estimated AAPT fulfilment rate therefore has limited precision and may not generalize to other settings, particularly given that 96.1% of participants were women. Second, AAPT classification was reconstructed retrospectively from records that were not originally designed for standardized AAPT assessment. The observed κ = 1.00 demonstrates reproducibility of interpretation of the available documentation, not validity or completeness of the source information. The depth of longitudinal documentation also varied among patients, with a look-back extending up to approximately 8–10 years in some cases. Fibromyalgia symptoms may fluctuate over time, including pain distribution, fatigue, and sleep disturbance. Consequently, retrospective classification cannot establish that an individual patient’s AAPT status remained stable throughout the disease course; some patients classified as AAPT NOT MET on the available documentation may have fulfilled the criteria at another time point.

Third, the concomitant-diagnosis endpoint was based on clinician-documented musculoskeletal or pain-related conditions rather than on a prospectively standardized confirmation protocol. Structural abnormalities may be incidental or only partly related to the presenting pain, and the association with AAPT status may reflect criterion-related overlap, referral bias, differential diagnostic ascertainment, and residual confounding. Fourth, no multivariable adjustment was performed. Given the modest sample size and the number of plausible, partly overlapping covariates, a post hoc adjusted model would have been vulnerable to instability and data-driven covariate selection. The reported OR should therefore be regarded as crude and descriptive.

Finally, the sampling frame combined patients referred for suspected fibromyalgia with patients carrying a previous clinician-assigned diagnosis, and these pathways could not be reliably separated for all patients. The 36.8% fulfilment estimate therefore applies only to the combined referral-based cohort and cannot be interpreted as a diagnostic confirmation or overdiagnosis rate among previously diagnosed patients. The exploratory symptom, medication, and comorbidity comparisons were also numerous relative to the sample size and were not adjusted for multiplicity; they require confirmation in adequately powered prospective studies.

## 5. Conclusions

In this combined referral-based cohort, approximately one-third of patients fulfilled the AAPT 2019 criteria on retrospective record-based adjudication. Documented concomitant musculoskeletal or pain-related diagnoses were common, although their higher frequency among AAPT MET patients may partly reflect criterion overlap, referral characteristics, and differential diagnostic ascertainment rather than an independent association. These findings support the complementary use of standardized classification and multidimensional pain phenotyping. Prospective, adequately powered studies using standardized assessment are required to confirm these observations. Generalizability is limited by the single-center design and predominantly female cohort.

## Data Availability

The data that support the findings of this study are available from the corresponding author upon reasonable request. The dataset contains pseudonymized clinical information from a single centre and cannot be made publicly available because the terms of the ethical approval and of patient consent do not cover open deposition.

## Supplementary Materials

The following supporting information can be downloaded at: https://www.mdpi.com/article/10.3390/healthcare14203478/s1, Table S1: Additional descriptive variables and exploratory between-group comparisons according to AAPT 2019 classification status; Table S2: Operational framework for retrospective AAPT adjudication from routine clinical records; Supplementary Analysis S1: DN4 missing-data sensitivity analysis.
