# Supplementary material for: AAPT Fibromyalgia Criteria and Concomitant Musculoskeletal Diagnoses in a Referral-Based Pain Clinic Cohort: A Retrospective Pilot Study
Source: Healthcare (Basel). 2026 Oct 16;14(20):3478. doi: 10.3390/healthcare14203478 (PMC13704118; doi:10.3390/healthcare14203478)
Supplement: Supplementary file 1 [file healthcare-14-03478-s001.zip › healthcare-4592345-supplementary.pdf]

Supplementary Table S1. Additional descriptive variables and exploratory between-group comparisons according to AAPT 2019 classification status

Variable	AAPT MET (n = 28)	AAPT NOT MET (n = 48)	p
Symptom scales (median [IQR])			
FIQR physical function (0–30)	16.2 [10.5–20.7]	11.7 [5.7–19.8]	0.097
FIQR overall health (0–20)	12.5 [9.0–16.0]	10 [4.0–15.0]	0.038
FIQR symptoms (0–50)	29.8 [26.2–32.5]	24.5 [19.5–30.5]	0.006
Zung SDS – emotional	17 [13.0–19.0]	13.5 [11.0–17.0]	0.026
Zung SDS – somatic	22.5 [20.8–25.2]	22 [17.8–23.2]	0.165
Zung SDS – cognitive	8 [7.0–9.2]	6.5 [5.0–8.0]	0.023
Self-reported medications, n (%)			
Tricyclic antidepressants	5 (17.9)	5 (10.4)	0.484
SNRI	5 (17.9)	6 (12.5)	0.521
SSRI	5 (17.9)	3 (6.2)	0.136
Other antidepressants	0 (0.0)	0 (0.0)	–
Gabapentinoids	8 (28.6)	8 (16.7)	0.252
Other anticonvulsants	0 (0.0)	1 (2.1)	1.000
Benzodiazepines	2 (7.1)	7 (14.6)	0.472
Z-drugs	2 (7.1)	2 (4.2)	0.623
Muscle relaxants	11 (39.3)	18 (37.5)	1.000
Paracetamol	6 (21.4)	20 (41.7)	0.085
NSAIDs	16 (57.1)	23 (47.9)	0.483
COX-2 inhibitors	7 (25.0)	6 (12.5)	0.210
Weak opioids	8 (28.6)	5 (10.4)	0.059
Strong opioids	0 (0.0)	3 (6.2)	0.293
Cannabinoids	1 (3.6)	0 (0.0)	0.368
Individual comorbidities, n (%)			
Anxiety/depression	6 (21.4)	5 (10.4)	0.310
IBS / functional GI	6 (21.4)	7 (14.6)	0.532
Headache / migraine	9 (32.1)	11 (22.9)	0.425
Sleep disorder	4 (14.3)	3 (6.2)	0.412
Thyroid disease	6 (21.4)	11 (22.9)	1.000
Raynaud phenomenon	1 (3.6)	2 (4.2)	1.000
Psoriatic arthritis	4 (14.3)	1 (2.1)	0.059
Other autoimmune disease	4 (14.3)	3 (6.2)	0.412

Continuous variables are presented as median [interquartile range (IQR)] and were compared using the two-sided Mann–Whitney U test; categorical variables are presented as n (%) and were compared using the two-sided Fisher’s exact test, which was preferred to the χ^2 test because expected cell counts were <5 in most comparisons. Medication categories are not mutually exclusive.

This table contains 28 exploratory hypothesis tests (6 symptom-subscale comparisons, 14 testable medication-class comparisons, and 8 comorbidity comparisons); one medication class had no events in either group and was not testable. No adjustment for multiplicity was applied. All comparisons in this table are exploratory and hypothesis-generating; individual p values should not be interpreted as confirmatory evidence and require confirmation in adequately powered prospective studies.

Medications reflect treatments reported by the patient in the pharmacological history recorded in the clinical chart. Information on timing, dose, duration, adherence, and treatment indication was not available, and current use could not be reliably distinguished from previous use.

AAPT, ACTTION–American Pain Society Pain Taxonomy; COX-2, cyclooxygenase-2; FIQR, Revised Fibromyalgia Impact Questionnaire; GI, gastrointestinal; IBS, irritable bowel syndrome; IQR, interquartile range; NSAID, non-steroidal anti-inflammatory drug; SDS, Zung Self-Rating Depression Scale; SNRI, serotonin–noradrenaline reuptake inhibitor; SSRI, selective serotonin reuptake inhibitor.

Supplementary Table S2. Operational framework for retrospective AAPT adjudication from routine clinical records

AAPT component	Clinical information reviewed	Operational approach in this study	Handling of insufficient/ambiguous documentation
Multisite pain (≥6/9 AAPT body regions)	Narrative pain history; anatomical pain-distribution descriptions; relevant clinical examination findings	Region involvement was assigned from explicit anatomical descriptions in the clinical record. Imaging abnormalities alone were not considered sufficient to establish pain in a region.	If the available record did not permit reliable reconstruction of pain distribution across the required regions, the record was considered insufficient for AAPT classification.
Fatigue or sleep disturbance of at least moderate clinical relevance	Clinician-documented fatigue and/or sleep disturbance in the routine clinical history	No retrospective numerical severity score was reconstructed. Assessors relied on the clinician-documented description of symptom severity and clinical relevance. FIQR fatigue and sleep items were not available during adjudication and did not determine AAPT status.	If documentation was too limited or ambiguous to support a reliable determination, the record was considered insufficient for AAPT classification.
Symptom persistence ≥3 months	Current and historical clinical documentation; longitudinal symptom history when available	Persistence was assessed using the available longitudinal record. The retrospective look-back varied by patient and extended approximately 8–10 years in some cases.	When duration could not be established reliably from the available documentation, the record was considered insufficient for AAPT classification.
Independent adjudication	AAPT-relevant clinical information only	Two clinical investigators independently applied the published AAPT 2019 framework and study definitions. No separate formal calibration exercise was performed. Assessors were blinded to each other's classification and to the non-AAPT variables specified in the Methods.	Five of 81 screened records were excluded for insufficient source information before the analytical phase. Complete agreement was observed among the 76 classifiable records (κ = 1.00), indicating reproducibility of record interpretation rather than validation of source-data completeness.

AAPT, ACTTION–American Pain Society Pain Taxonomy; FIQR, Revised Fibromyalgia Impact Questionnaire. This table summarizes the study operationalization of retrospective AAPT adjudication and does not represent a prospectively validated diagnostic protocol.

Supplementary Analysis S1. DN4 missing-data sensitivity analysis

DN4 data were available for 67/76 patients (24/28 AAPT MET and 43/48 AAPT NOT MET). The complete-case comparison showed DN4 positivity (score ≥4) in 22/24 AAPT MET patients (91.7%) and 26/43 AAPT NOT MET patients (60.5%). Because nine observations were missing, post hoc sensitivity analyses examined allocations of the four missing AAPT MET observations and five missing AAPT NOT MET observations. The result was not robust to plausible missing-data allocations.

These post hoc analyses demonstrate that the nominal complete-case DN4 finding is sensitive to assumptions regarding missing observations. DN4 is a screening instrument for neuropathic-like symptoms and signs and should not be interpreted as establishing neuropathic pain or a distinct neuropathic pain phenotype.

**Scenario**	**AAPT MET DN4-positive**	**AAPT NOT MET DN4-positive**	**Statistical result**
Complete-case analysis	22/24 (91.7%)	26/43 (60.5%)	OR 7,19; Fisher p = 0,010.
Tipping-point allocation: all 4 missing AAPT MET values negative; 1 of 5 missing AAPT NOT MET values positive	22/28 (78.6%)	27/48 (56.3%)	OR 2.85; two-sided Fisher's exact p = 0.081
Most adverse allocation: all 4 missing AAPT MET values negative; all 5 missing AAPT NOT MET values positive	22/28 (78.6%)	31/48 (64.6%)	OR 2.01; two-sided Fisher's exact p = 0.301
